# Changes in brain structure and function in youth at familial risk for schizophrenia or bipolar disorder: implications for early intervention

**DOI:** 10.1192/j.eurpsy.2024.46

**Published:** 2024-08-27

**Authors:** G. Sugranyes, BASYS Working Group

**Affiliations:** ^1^Department of Child and Adolescent Psychiatry and Psychology, Hospital Clinic Barceolona; ^2^Clinical and Experimental Neuroscience, FCRB-IDIBAPS , Barcelona, Spain

## Abstract

**Abstract:**

The evaluation of child and adolescent offspring of patients with schizophrenia or bipolar disorder seeks to understand changes taking place in the brain in individuals at heightened risk for disease during a key developmental period. In this session I will present findings from the BASYS (Bipolar And Schizophrenia Young offspring Study) cohort, which has recruited young offspring of patients with schizophrenia or bipolar disorder ages 6 to 17 years, using clinical, cognitive and brain imaging measures for over 15 years in Spain. I will begin by reviewing our baseline and 2 year findings using structural magnetic resonance imging (MRI) measures, where we found whole brain and regional cortical grey matter volume and surface area reductions, specifically in offspring of patients with schizophrenia relative to controls, but not in offspring of patients with bipolar disorder, which I will compare with results from the ENIGMA relatives working group analyses. Within our cohort I will expain the relevance of baseline brain structural findings to clinical and cognitive outcome over time. I will then present longitudinal analyses of structural anf functional MRI measures at up to 8 year follow-up, examining the influence of development of psychotic spectrum symptoms over time and cognitive and functional outcomes, on longitudinal brain imaging measures. I will finish the talk explaining avenues for future research in the field, which include incorporating other imaging modalities and validating our findings in other cohorts, while I will also present avenues for increasing understanding of the neurobiological changes underpinning our MRI findings.

This project has received funding from Instituto de Salud Carlos III (PI151500467; PI1700741; PI1800696; PI1800976, PI2100330), Fundació Marato TV3 (091630, 202232-30-31), the Catalonia Government (2021SGR01319), PERIS (SLT006/17/00346), Fundació Clínic Recerca Biomèdica (Ajut a la Recerca Pons Bartran), co-financed by ERDF Funds from the European Commission and CIBERSAM.

**Disclosure of Interest:**

None Declared

